# Biodegradable Polymer Materials Based on Polyethylene and Natural Rubber: Acquiring, Investigation, Properties

**DOI:** 10.3390/polym14122457

**Published:** 2022-06-16

**Authors:** Ivetta Varyan, Polina Tyubaeva, Natalya Kolesnikova, Anatoly Popov

**Affiliations:** 1 Joint Research Center, Plekhanov Russian University of Economics, 36 Stremyanny Lane, 117997 Moscow, Russia; polina-tyubaeva@yandex.ru (P.T.); anatoly.popov@mail.ru (A.P.); 2 Department of Biological and Chemical Physics of Polymers, Emanuel Institute of Biochemical Physics, Russian Academy of Sciences, 4 Kosygina Street, 119334 Moscow, Russia; kolesnikova@sky.chp.ras.ru

**Keywords:** natural rubber, biodegradation, polyethylene, supramolecular structure, wettability, exposure in soil

## Abstract

The growing amount of synthetic polymeric materials is a great environmental problem that has to be solved as soon as possible. The main factor aggravating this problem is the abundance of products made from traditional synthetic polymer, such as packaging materials, cases, containers and other equipment with a short period of use, which quickly turns into polymer waste that pollutes the ecosystem for decades. In this paper, we consider the possibility of solving this problem by the development of biodegradable compositions based on polyolefins and elastomers. The addition of a natural component (natural rubber) to the matrix of the synthetic polymeric (polyethylene) leads to the significant changes in structure and properties of the material. Different aspects of mixing semicrystalline and amorphous polymers are discussed in the article. It was shown that addition of 10–50% wt. of the elastomers to the synthetic polymer increases wettability of the material, slightly reduces the mechanical properties, significantly affects the supramolecular structure of the crystalline phase of polyethylene and initiates microbiological degradation. In particular, in this work, the acquisition, structure and properties of biodegradable binary composites based on low-density polyethylene (LDPE) and natural rubber (NR) were studied. It has been shown that such compositions are biodegradable in soil under standard conditions.

## 1. Introduction

Currently, an important environmental problem is the ever-increasing amount of waste of synthetic polymeric materials both on land and at sea. The spread of plastic pollution correlates with the low price and durability of plastics, as well as the indispensability of this material at the moment in some areas, which determines the high level of its use by humans [1].

Products made of polymer materials such as packaging materials, cases of house-hold and other equipment, containers, disposable medical products and utensils, quickly turn into polymer waste, polluting the environment. At the same time, it is worth noting that up to 90% of all plastic ever produced in the world still exists, and of this amount, 60–70% is municipal solid waste in the form of plastic packaging [2].

Plastic waste in all forms is harmful to nature and living beings. Toxic substances can be released from polymers, causing the death of plants and animals both on land and in water, and can also cause diseases in humans. Accumulating in large quantities, plastic waste is a threat to entire ecosystems, especially near water bodies and rivers, as well as in the waters of the seas and oceans. Plastic pollution also harms the economy—the fishing industry suffers significant losses and the construction of treatment facilities and the development of plastic recycling methods require substantial financial investments. In addition, the tourism industry is also suffering, since plastic household waste dumps near recreational areas are not only unattractive but can also be a source of unpleasant odors and toxic fumes.

The separation of polymeric materials from soil or other pollutants is a laborious and energy-intensive process. It is not always possible to use these materials in recycling [3]. According to a number of experts [4], a radical solution to this problem is the creation of polymers capable of undergoing biodegradation after operation under appropriate conditions with the formation of carbon dioxide and water, which are nontoxic to plants [5].

Biodegradable polymers are materials with an adjustable service life. Such polymers spontaneously decompose as a result of natural microbiological and chemical processes [6]. The term “biodegradable” is commonly used to refer to a polymer whose destruction or deterioration of its former qualities can be caused at least partially by a biological system [7]. As a rule, polymer biodegradation is initiated not only by biological processes but also by the absorption of heat or light by the polymer, mechanical damage, chemical reactions or diffusion of environmental components into the material, etc., which ultimately can lead to material degradation and increased attack by microorganisms [8].

There are a number of characteristics of polymers that affect biodegradation during composting and attractiveness for microorganisms. The most important are the chemical nature of the polymer, the molecular weight, the structure of its molecules, the branching of the macrochain (the presence and nature of side groups) and the supramolecular structure [9]. Polymers with an amorphous supramolecular structure are always less resistant to biodegradation than crystalline ones. The resistance of polymeric materials to the action of microorganisms also depends on the plasticizers, fillers, stabilizers, and other techno-logical additives included in their composition, as well as on the extent to which these substances can be a source of carbon, nitrogen, and other biogenic elements for micro-organisms [10]. It is known that inorganic components (silicates, sulfates, phosphates, carbonates) do not support the growth of microorganisms [11].

Biodegradable polymers were developed several decades ago, but their full-scale commercial application has been very slow. This was because they were generally more expensive and had less stable physical properties than traditional plastics [12]. New large-scale production systems reduce the cost of producing biodegradable polymers, while advanced polymerization and blending technologies make these materials stronger and more durable [13].

The most important function of biodegradable polymers is the imparting of biodegradability properties to large-capacity industrial polymers, among which are polyolefins (polyethylene, polypropylene, etc.) [14]. Polyolefins are high-molecular compounds that are produced from oil and natural gas by polymerization of low-molecular substances—olefins [15]. An important factor, as noted above, which determines the resistance of a polymer to biodegradation, is the size of its molecules. Monomers affected by microorganisms serve as a source of carbon for them, while polyolefins with a large molecular weight are quite resistant to the action of microorganisms [16]. It is necessary to activate the degradation processes in the material, which will lead to a decrease in the molecular weight of the polymer and the appearance of low-molecular-weight, bioassimilable parts. A promising area of research in this area is the creation of composites based on synthetic polymers and biodegradable natural fillers, the addition of which to the matrix of a synthetic polymer makes it possible to obtain materials that can rapidly decompose under environmental conditions [17,18,19].

Synthetic polymers have high mechanical and thermal characteristics but are resistant to the action of microorganisms and are not capable of degradation, whereas natural polysaccharides, although biodegradable, have poor mechanical parameters. In order to maximize the use of the properties of each of the components, natural components (starch, chitin, cellulose, amylose, amylopectin, dextrin, etc.), which are a nutrient medium for microorganisms, are used as additives to synthetic polymers [20,21,22,23]. The polymer composite material obtained from such a mixture can be called biodegradable, since the synthetic polymer matrix in this case decomposes into bioassimilable fragments.

Vinyl ketone monomers are photoinitiators of the decomposition of polyethylene and polystyrene. The introduction of such substances in a small amount as a copolymer to ethylene or styrene makes it possible to obtain plastics with properties similar to polyethylene or polystyrene, but capable of photodegradation [24,25]. In order to accelerate photodegradation and subsequent biodegradation of polyolefins, alkyl ketones, cellulose, or fragments containing carbonyl groups are introduced into them [26,27].

The use of chitin and chitosan as fillers is known [28,29]. The shells of crustaceans and insects are the main source of chitin, from which, in turn, chitosan is obtained. Due to biocompatibility with human tissues of low toxicity, the ability to enhance regenerative processes during wound healing and biodegradability, materials based on chitosan are of particular interest for medicine [30].

When creating biodegradable polymer compositions based on synthetic polymers and starch, a high content of the latter is usually required, which inevitably leads to a deterioration in the technological and operational characteristics of finished materials due to poor distribution of components in the polymer matrix [31,32].

Since biodegradation is influenced by a large number of factors (temperature, pressure, humidity, salt concentration, presence or absence of oxygen, pH, stability of environmental conditions, etc.), it is difficult to predict the behavior of biodegradable materials and the exact timing of complete biodegradation [33,34,35,36,37].

In addition, it is obvious that to increase the production of biopolymers and com-posites based on them, significant economic costs are required and significant agricultural areas and resources are required for growing raw materials.

The study of the processes of destruction of materials based on polyolefins with various additives under the influence of various aggressive factors is an urgent scientific and practical task.

It should be noted that the addition of natural fillers to a synthetic polymer matrix can significantly affect not only biodegradability, but also other properties of materials. In this case, it is possible to obtain polymers with improved mechanical or thermal properties. At the same time, the use of natural fillers obtained from the waste products of agricultural production can significantly reduce the cost of such materials, which makes their profitability very high. So, for example, in the work [38,39], composite materials based on low-density PE, filled with fibers obtained from corn husks, were studied. In particular, the effects of fiber components on the mechanical, thermal, water absorption, and crystalline properties of reinforced PE/corn hull fiber composites were studied. However, such materials have worse performance properties compared to those made with the addition of elastomers.

There are also a number of works describing polymeric materials with the addition of oxo-degradable additives based on transition metal salts of cobalt, nickel, or iron. Such additives are widely used to make polyolefins biodegradable [40,41,42,43,44,45]. Under natural conditions, the decomposition of such materials occurs in two stages. At the first stage, under the action of sunlight and oxygen, the plastic product breaks down into fragments. At the second stage, complete or partial decomposition of plastic fragments occurs due to the vital activity of microorganisms. Note that when recycling waste in real conditions, the simultaneous presence of all factors necessary for the implementation of the first stage of plastic decomposition is difficult to achieve. Therefore, studies show that over a period of 350 days, only about 15% of oxo-degradable LDPE placed in soil degrades to carbon dioxide [46].

One of the research directions of polymer composite materials is the development of biodegradable compositions based on polyolefins and elastomers. As an additive to synthetic polymeric materials, it is proposed to use elastomers—for example, rubber (natural or synthetic), a product of plant origin—products that are quickly subjected to oxidative degradation and microbiological destruction. Natural rubber (NR), found in the milky sap of rubber plants such as Brazilian hevea and dandelion, is an extremely important natural high-molecular-weight hydrocarbon, isoprene cispolymer, characterized by elasticity, water resistance and electrical insulating properties.

Dual composites based on low-density polyethylene (LDPE) and NR show fairly good physical and mechanical properties and are biodegradable.

In the field of biodegradable plastics, there is a constant increase in the production capacity of polymeric materials already in demand, as well as the development and ex-pansion of the range of new compositions that are characterized by environmental friendliness, the possibility of modifying the required specified service life and the ability to biodegrade without harm to the environment. In this regard, the range of developed biodegradable additives is also expanding.

The most efficient and cost-effective direction of work in the field of creating bio-degradable polymers based on synthetic macromolecular compounds is the mixing of synthetic and natural biodegradable polymers. In modern research, from our point of view, an important problem is to impart biodegradability properties to large-tonnage traditional polymers (polyethylene, polypropylene, polyvinyl chloride, etc.), the production of which has undeniable advantages and is currently more environmentally friendly and energy- and resource-saving. One of the promising areas of research on polymer composite materials is the development of biodegradable compositions based on polyolefins and elastomers, in particular, polyethylene and natural rubber.

Thus, one of the promising areas of research to solve this problem is the creation of polymeric materials, the utilization of which is possible under the influence of the environmental microbiota [47]. For this purpose, natural components are used as additives to synthetic polymers, which are a nutrient medium for microorganisms [48].

Due to their large molecular weight, polyolefin molecules cannot be easily assimilated by microorganisms. The hydrophobic nature of polyolefins limits the effect of microbial enzymes on them, and the stabilizers contained in industrial polymers prevent oxidation during processing and degradation [49,50]. In this regard, there is a need to modify the polyethylene matrix in such a way that, while maintaining the main operational properties of the product, it would be possible to dispose of it after the expiration of its use under the influence of the environmental microbiota. Evaluation of the effect of small additions of natural rubber, which biodegrades rather quickly in the soil, on the properties of such a large-tonnage polymer as low-density polyethylene (LDPE) is of both scientific and practical interest.

The addition of natural rubber (NR) to the matrix of a synthetic polymer (polyethylene) leads to significant changes in the structure and properties of the material. To address this issue, it is necessary to study various aspects of mixing semi-crystalline and amorphous polymers to study the physical and mechanical properties of materials, surface hydrophilicity and biodegradation of composites as a result of composting in soil. But despite the fact that there is a large amount of research on the creation of films for agriculture, there are still not enough of these materials on the market [49,50,51]. Thus, the purpose of our work was to study the structure of mixtures based on polyethylene and natural rubber to evaluate their biodegradation and to analyze the properties of such polymer composites that can be used in agriculture, packaging and other areas of light industry.

## 2. Materials and Methods

### 2.1. Materials

The objects of the study were compositions of low density polyethylene brand 15803-020 (LDPE, Neftekhimsevilen, OJSC, Kazan, Russia) with natural rubber (NR, grade SVR 3L, Dong Xoai, Vietnam).

**Figure 1:**
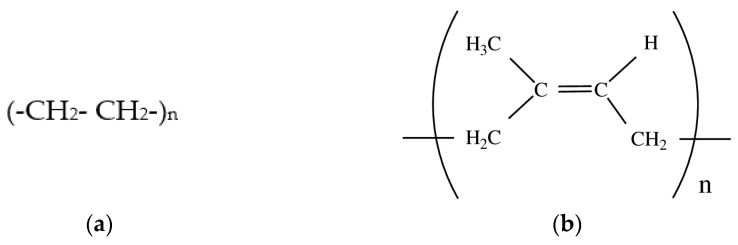
Structural formulas of LDPE (**a**) and natural rubber (**b**).

Low density polyethylene: molecular weight 2 × 10^5^; density 0.9190 ± 0.002 g/cm^3^; melt flow index 2.0 ± 0.5 g/10 min.; the structural formula is shown in Figure 1a.

Polyolefins are the most common type of polymers obtained by polymerization and copolymerization of unsaturated hydrocarbons (ethylene, propylene, butylene, etc.). About 50% of the ethylene produced in the world is used to produce polyethylene.

Ethylene can be polymerized in several ways. Depending on this, polyethylene is divided into: high pressure polyethylene or low density polyethylene (LDPE); low pressure polyethylene or high density polyethylene (HDPE); and also linear polyethylene [52].

LDPE is a thermoplastic polymer obtained by the polymerization of ethylene monomer under the action of high temperatures (up to 1800 °C), pressure up to 3000 atmospheres, and with the participation of oxygen [53,54]. LDPE is a lightweight, durable, elastic material used in many areas of activity of a modern person. LDPE has relatively weak intramolecular bonds and therefore a lower density than other types of polymers.

In their structure and properties (despite the fact that the same monomer is used), LDPE, HDPE and linear polyethylene differ and, accordingly, are used for various tasks. LDPE is a soft material; HDPE and linear polyethylene have a rigid structure. Differences also appear in density, melting point, hardness and strength. The properties of LDPE are largely determined by the degree of branching, which is characterized by the number of branches per 1000 carbon atoms [52].

Natural rubber: molecular weight 1.25 × 10^6^; density 0.92 g/cm^3^; the structural formula is shown in Figure 1b.

### 2.2. Preparation of Materials

Compositions of LDPE with NR were prepared on a Brabender-type laboratory rotary mixer. The total sample weight was 25 g. The mixing process was carried out at a temperature of 140 °C in an argon atmosphere. At a rotor speed of 15 rpm, crushed rubber was loaded into the mixing chamber. After 2 min, polyethylene granules were added, the speed of rotation of the rolls was increased to 45 rpm and mixing was continued for 10 min. After mixing, the mixtures were removed from the mixing chamber and cooled to room temperature. The compositions of the LDPE/NR blends are given in Table 1.

Film samples were obtained from crushed mixtures on a laboratory hydraulic press with an electronic unit for heating plates at a temperature of 140 °C and a pressure of 60 kgf/cm^2^ on a cellophane substrate. The crushed samples of the mixtures were evenly distributed on the substrate. The duration of pressing was 2 min. Next, the obtained samples were rapidly cooled in water at a temperature of about 240 °C. The thickness of the obtained samples was 130 ± 10 µm.

### 2.3. Methods

#### 2.3.1. Contact Angle

It was of interest to determine the degree of hydrophilicity of the surface of the samples depending on the content of natural rubber in the mixture. The hydrophilicity of the films was judged from the results of measuring the internal contact wetting angle formed between the water drop and the sample surface. The contact angle was measured using an optical microscopeOlympus BX43 (Olympus, Tokyo, Japan) (objective FMA050 magnification ×50. Image processing was made using Altami Studio 3.4 (Altami, Saint-Petersburg, Russia).

Water drops (2 μL) were applied to three different areas of the film surface using an automatic dispenser. The result is the average of three measurements from different parts of the sample. The relative measurement error ranged from 0.5 to 2%.

#### 2.3.2. Differential Scanning Calorimetry

Determination of thermophysical parameters of LDPE melting (enthalpy and melting temperature) in mixtures with NR was carried out using scanning calorimetry (DSC) by the Netzsch 214 Polyma (Netzsch, Selb, Germany). A sample weighing about 10 mg was tightly sealed in an aluminum container and placed in the cell of the calorimetric chamber. An empty container equal in weight to within 0.5 mg was placed in the comparison cell. Melting endotherms were recorded in the temperature range 40–140 °C at a heating rate of 8°/min. To calibrate the temperature and enthalpy of fusion, a standard sample of indium (In) was used (specific heat of fusion ∆*N*_sp_ = 28.4419 J/g; *T*_m_ = 156.6 °C). The melting temperature was determined from the maximum of the endothermic melting peak of the sample, the heat of fusion, according to the area of the melting peak, limited by the base line.

(1) $$\chi =\frac{\Delta H}{H_{\mathrm{LDPE}}}\times 100\%$$

where ΔH is melting enthalpy; $H_{\mathrm{LDPE}}$ is the melting enthalpy of the ideal crystal of the LDPE, 293 J/g [55].

The length of the lamellae was estimated by the formula:

(2) $$T_{m\;}=T_{m\mathrm{LDPE}}\;\left(1-\frac{2\gamma }{\Delta H\times l}\right)$$

where *T*_m_ is the melting point of the sample, ∆*H* is melting enthalpy, *T*_mLDPE_ is the equilibrium melting point of the ideal crystal of the LDPE, equal to 128.85 °C [55], γ is the top and bottom fold surface free energy, *l* is the lamellar thickness, and Δ*H* is the heat of fusion per cubic centimeter of the perfect crystal. The well-known value of γ is 0.09 J m^−2^ for LDPE, which was obtained from polymer nucleation theory [55].

#### 2.3.3. Mechanical Analysis

The main indicator of consumer properties of the material is its mechanical characteristics. The mechanical characteristics of film materials are determined by the parameters of tensile strength and relative elongation at break.

Determination of the strain–strength properties of film materials under tension was carried out using a tensile testing machine, GPUG5 DLC-0,5 (DVT Devotrans, Istanbul, Turkey). The stretching speed was 100 mm/min. Samples were cut from films whose working length was 40 mm. The samples were cut in such a way that they did not have burrs along the edges or defects on the surface. The number of test samples for each type of mixture was 5. Tensile strength and maximum relative elongation at break were determined from the tensile diagrams.

#### 2.3.4. Biodegradation

The soil is the main habitat for microorganisms. The most saturated layer of soil is at a depth of 5–15 cm (aerated layer), 1 g of which contains up to 108 units of microorganisms. In addition, as a rule, the more organic residues in the soil, the more microorganisms it contains [54].

To study the biodegradation of the tested materials, a soil test was carried out using reconstituted soil simulating real soil. With the help of regular watering and measurement, the moisture capacity of the soil was maintained at the level of 60%, which is optimal for the biological activity of microorganisms. Film samples were vertically immersed in the center of the soil volume and kept at room temperature (22 ± 3 °C) for certain time intervals.

After the expiration of time, the samples were removed from the soil, cleaned of the soil, and brought to a constant weight in air. After that, a visual assessment (color change, loss of transparency) of the composites was carried out, as well as an analysis of the change in the mass of the samples.

Determination of biodegradation during composting of samples was determined by the change in mass of samples based on the following equation.

(3) $$\Delta m_{i}=\frac{m_{H}-m_{i}}{m_{H}}\times 100\%,$$

where Δ*m*_i_ is relative weight loss; *m*_H_ is sample initial weight and $m_{i}$ is mass of the sample after soaking it in the soil.

#### 2.3.5. Microscopy

Determination of the degree of uniformity of the distribution of the natural rubber phase in the polyethylene matrix, as well as the degree of micromycete development on the surface of the samples, was carried out using an Olympus BX43 (Olympus, Japan, Tokyo) optical microscope at a magnification of 200× g in the transmitted light mode. The images were recorded on microphotographs.

## 3. Results

### 3.1. Distribution of Natural Rubber Particles in the Polyethylene Matrix

The mixing of polymers such as polyethylene and natural rubber is characterized by thermodynamic instability of the system, due to significant differences between the structural organization and properties of semi-crystalline PE and amorphous NR. The mixing of such components affects the kinetics of the crystallization processes of the semi-crystalline component [56], which largely determines the properties of the composite. This approach is used in hardening, to control the phase structure, to increase the impact strength and for directed modification of polymer composites [57].

**Figure 2:**
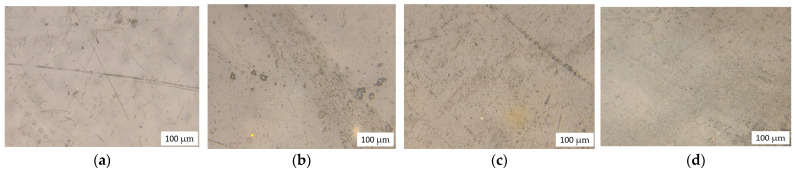
The microphotographs of initial samples: LDPE 100 (**a**), LDPE/NR 90/10 (**b**), LDPE/NR 70/30 (**c**), LDPE/NR 50/50 (**d**).

In this regard, it is of great interest to study the nature of the distribution of rubber particles in the LDPE matrix. In order to determine the uniformity of the distribution of the natural rubber phase in the polyethylene matrix, the surface of the samples was examined using optical microscopy. Figure 2 shows micrographs of the initial samples of the surface of the samples at a magnification of 200 times.

It was found that rubber has characteristic features of distribution in the LDPE structure. At low concentrations, regardless of the quality and duration of mixing, rubber forms large particles in the LDPE matrix (Figure 2a). Moreover, with an increase in the concentration of NR, the average size of such inclusions decreases (Figure 2b,c), reaching a minimum at a concentration of LDPE/NR 50/50 (Figure 2e). Thus, being distributed in the LDPE structure, rubber tends to form conglomerates, the average size of which is given in Table 2.

**Figure 3:**
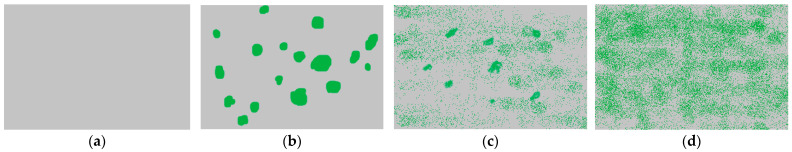
Schematic structure of the initial samples: LDPE 100 (**a**), LDPE/NR 90/10 (**b**), LDPE/NR 70/30 (**c**), LDPE/NR 50/50 (**d**), the LDPE matrix is shown in gray, the NR inclusion is green.

At equal concentrations of NR and LDPE, the average sizes of rubber particles reach minimum values and are difficult to distinguish, since they represent a network-like system. A schematic representation of the systems is shown in Figure 3.

### 3.2. Mechanical Properties

The introduction of fillers into polymers is accompanied by the formation of a new set of composition properties. The adsorption or the molecular interactions are responsible for adhesion at the interface, physical, mechanical and other properties of filled systems. Interfacial interactions determine the structural features of the boundary layer, the nature of the molecular packing, molecular mobility, morphology, and other properties [58,59,60,61,62,63,64]. Since mixed film samples were used in the work, it was of interest to study their strain–strength properties under tension. The properties of polymer mixtures, including mechanical ones, are determined by their structure and the variety of physical and chemical interactions of components at the interface. The influence of the composition of PE/NR mixtures on such parameters as tensile strength and maximum relative elongation was studied. The results are presented in Table 3.

Despite the fact that rubber has high elastic properties, its contribution to the properties of LDPE/NR composites leads to a decrease in strength by 50–74% and to a decrease in relative elongation by 45–80%. Moreover, the most interesting result is the change in relative elongation, since this indicator changes non-linearly, reaching an extreme at a concentration of 90/10.

### 3.3. Contact Angle

**Figure 4:**
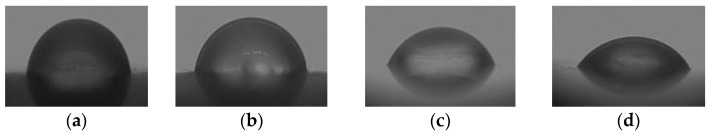
Contact angle measurement results. LDPE 100 (**a**), LDPE/NR 90/10 (**b**), LDPE/NR 70/30 (**c**), LDPE/NR 50/50 (**d**).

The initial stage of polymer biodegradation is the attachment (adsorption) of microorganisms to the polymer surface. The surface of LDPE is generally hydrophobic. Most microorganisms can attach to a surface if it is hydrophilic. It was of interest to determine the degree of hydrophilicity of the surface of the samples depending on the content of NR and additives in the mixture. The hydrophilicity of the films was estimated based on measurements of the contact angle formed between the water drop and the sample surface. Water drops were applied to three different regions of the film surface. The result is the average of three measurements from different sites. The relative measurement error ranged from 0.5 to 2%. Figure 4 show the results.

**Figure 5:**
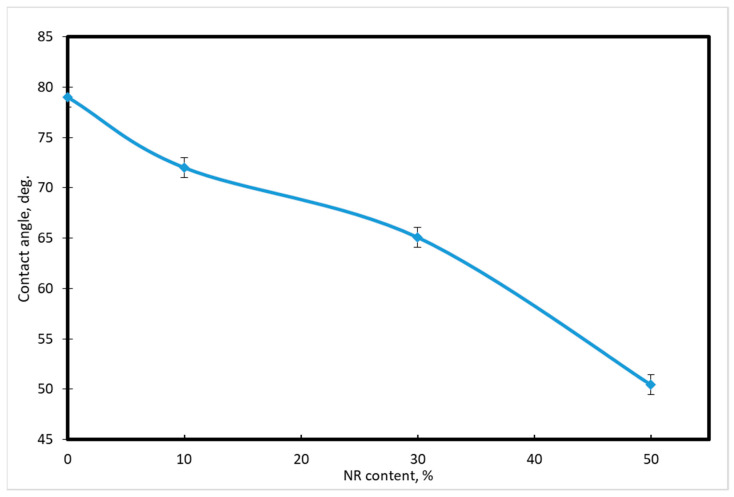
Change of contact angle depending on the content of natural rubber.

Values of the inner contact angle depending on the concentration of NR are shown in Table 4 and the dynamics of the changes can be seen in Figure 5. Thus, the contact angle increases by 35%, which ensures high hydrophilicity of the composite surface.

### 3.4. Differential Scanning Calorimetry

The study of the supramolecular structure of the obtained composites is of great interest. Using the DSC method in a nonisothermal mode, the process of melting of LDPE/NR samples with different ratios of components was studied. When samples of different compositions are heated, all thermograms show one endothermic peak in the temperature range corresponding to the melting of LDPE crystals. The DSC results of the samples are presented in Table 5.

**Figure 6:**
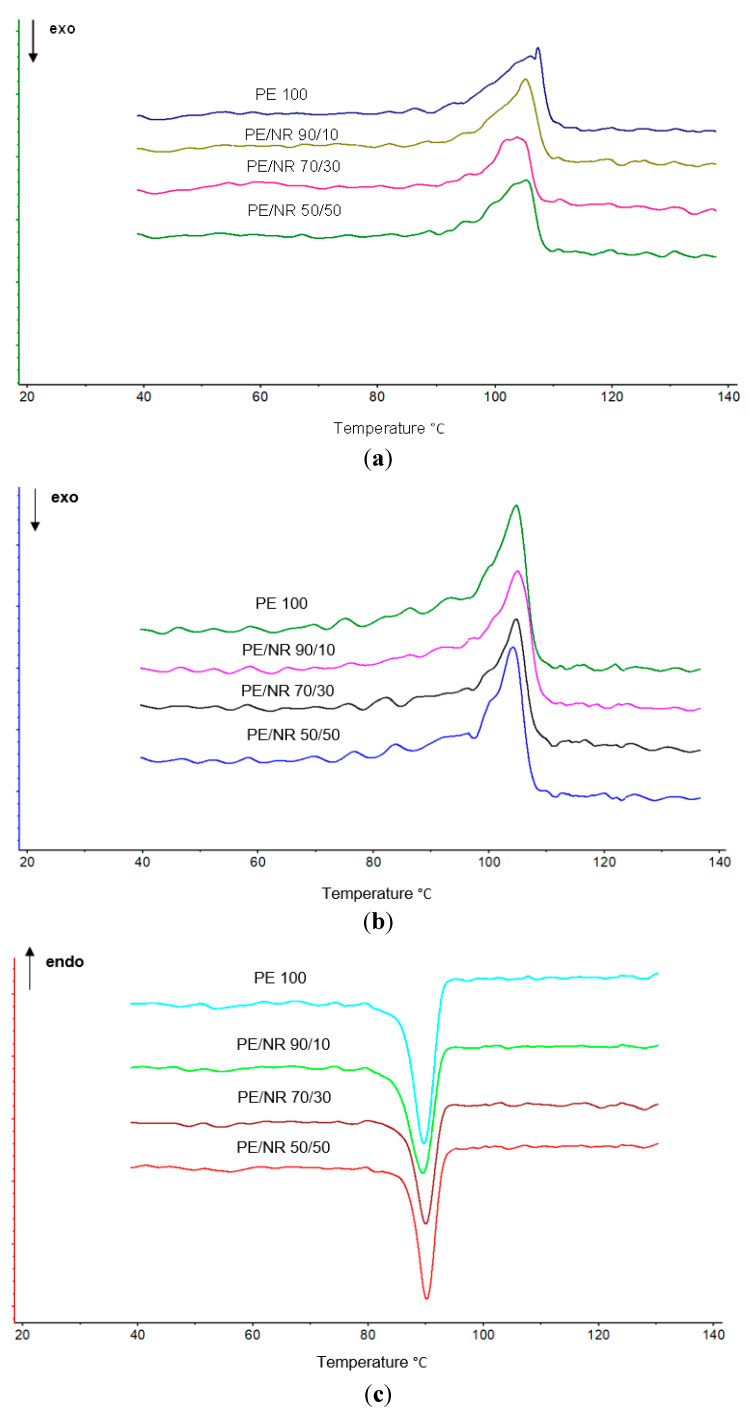

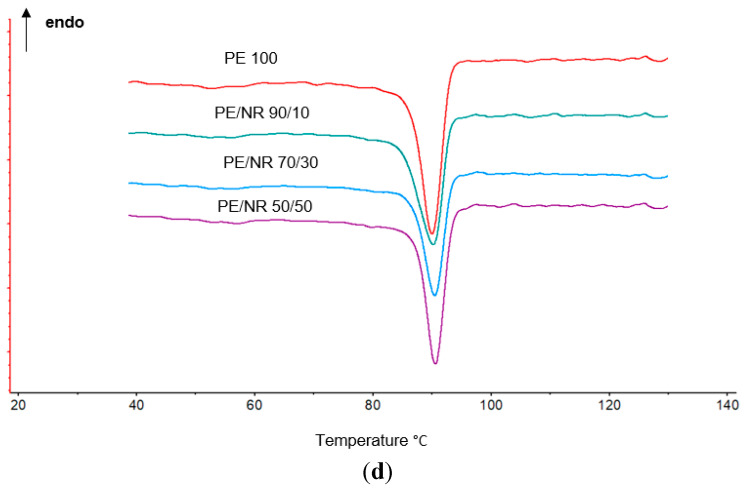
Melting peaks of initial samples: first heating (**a**), second heating (**b**). Cooling peaks of initial samples: first heating (**c**), second heating (**d**).

As can be seen from the table, the melting temperature varies within the measurement error. This fact indicates that the size of crystallites changes little, since their melting occurs at a similar temperature. However, significant changes are observed for the enthalpy of melting. NR has a significant effect on the process of LDPE crystallization, reducing the melting enthalpy by more than 40%. Of particular interest is also the shape of the melting and crystallization peaks (Figure 6).

At the first heating, we see the melting of the structure of the polymer subjected to wear. However, on the second heating, we already see the melting of the native polymer structure. And if on the second heating the initial LDPE has a higher degree of crystallinity than on the first one, then in the case of LDPE/NR composites, we also observe a decrease in the degree of crystallinity, but by 65%. This confirms that NR prevents LDPE crystallization, although on the cooling thermograms (Figure 6c,d) we see a fairly clear crystallization process.

### 3.5. Biodegradation

**Figure 7:**
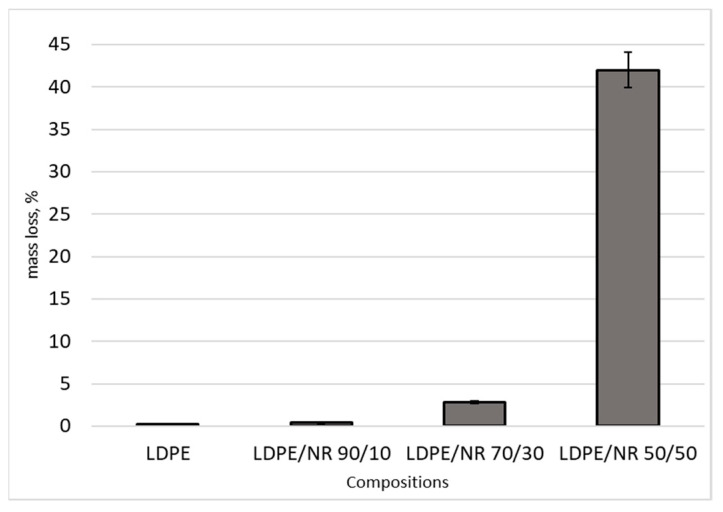
Sample weight loss over 24 months: LDPE 100 and LDPE/NR composites with NR content of 10, 30, 50 wt%.

Laboratory soil was also used for a comprehensive study of the biodegradation of the samples. The laboratory soil simulates the real soil while minimizing the difference between different soil types, achieving high reproducibility of results. The soil was prepared according to the standard method and consisted of equal amounts by weight of garden soil, sand and horse manure. Film samples were kept in the soil for 24 months. Figure 7 shows the mass loss of samples of different compositions after exposure in soil.

Figure 7 shows a significant increase in weight loss with an increase in the content of NR in the mixture. Compared to all samples, the LDPE/NR (50/50) sample shows the highest weight loss *(*Figure 7*).*

**Figure 8:**
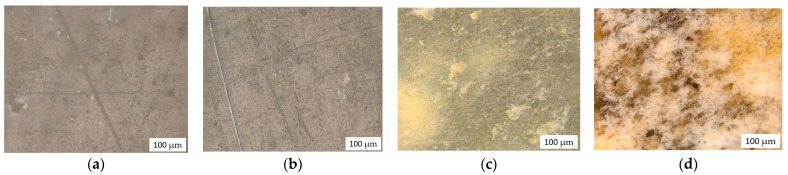
The microphotographs of samples after exposure in the soil: LDPE 100 (**a**), LDPE/NR 90/10 (**b**), LDPE/NR 70/30 (**c**), LDPE/NR 50/50 (**d**).

Figure 8 shows micrographs of samples after exposure in the soil for 24 months.

Samples with a content of 30% and 50% have the greatest visible changes compared to the rest (Figure 8c,d). Dark spots and staining are the result of exposure to soil microorganisms and their metabolic products. The microphotographs show that the original flat surface of the sample after exposure to the soil becomes stained with the products of the vital activity of microorganisms and also becomes uneven and loose, which indicates an active process of biodegradation. The question remains whether only NR is destroyed in the PE structure or whether LDPE also undergoes destruction. DSC results (Table 6) showed changes in the PE structure.

**Figure 9:**
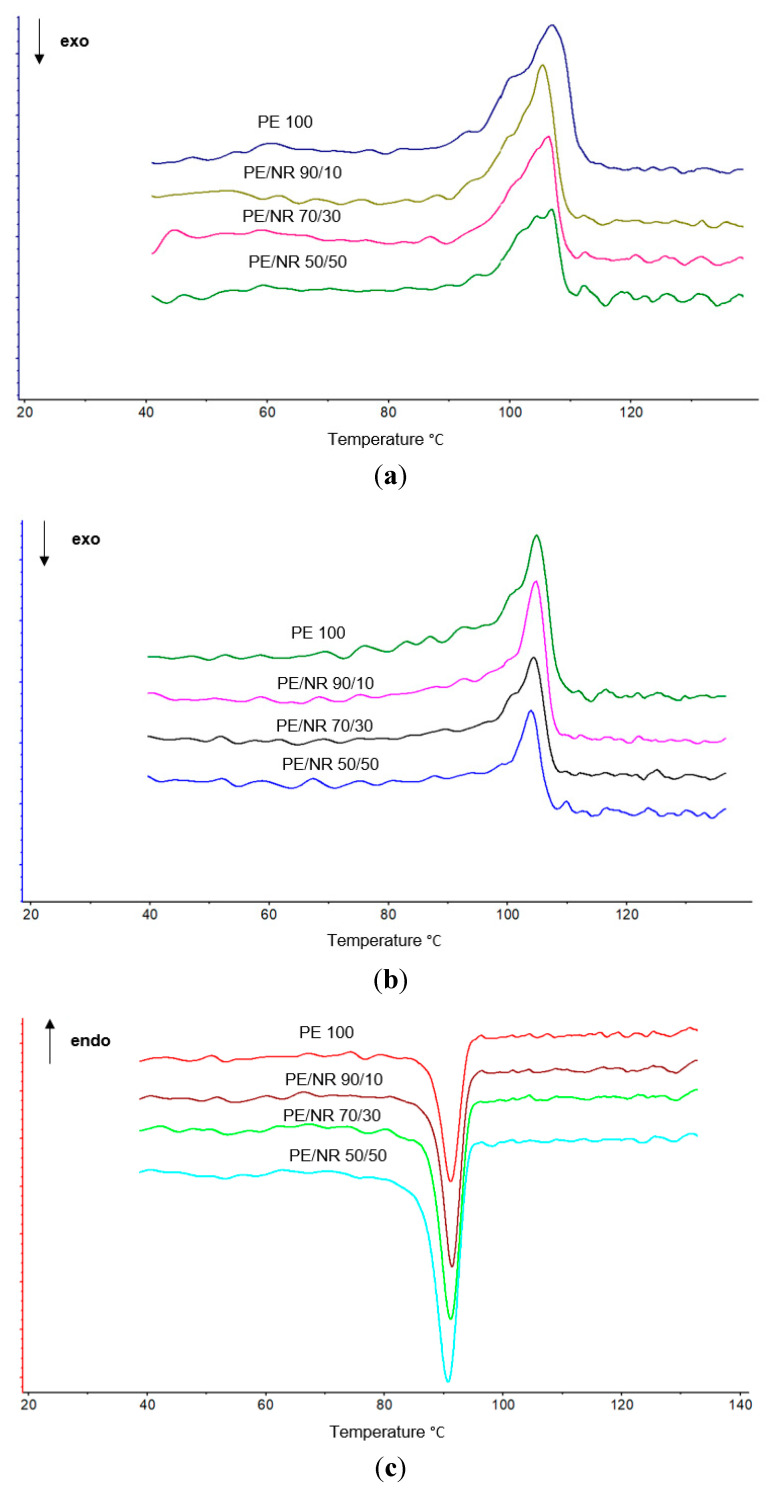

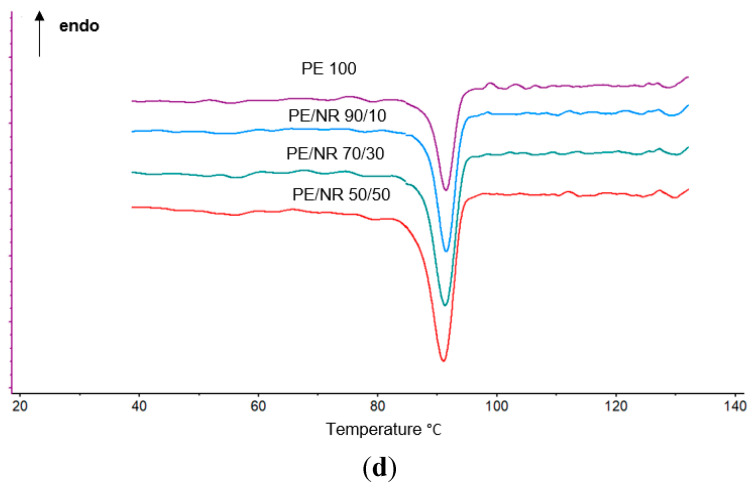
Melting peaks of samples after exposure in the soil: first heating (**a**), second heating (**b**). Cooling peaks of initial samples: first heating (**c**), second heating (**d**).

Thus, the crystallinity of LDPE increases after exposure in the ground: for the initial LDPE, LDPE/NR 90/10 and LDPE/NR 70/30 increased by 3%, and in the case of LDPE/NR 50/50, by 6%. On the second heating, the greatest results are visible for samples of LDPE/NR 50/50, for which the most intense biofouling occurred. The broadening of the shape of the peak of the second heating (Figure 9b) as well as the appearance of low-temperature arms in the region of 80–90 °C indicates oxidative degradation of PE, which NR significantly accelerates.

The change in the size of the lamellae is evidence of the biodegradation process. To confirm that the biodegradation process destroys not only NR but also LDPE macromolecules, an indirect assessment of the length of the size of the LDPE lamellae was carried out. A decrease in the length of the lamellae indicates a decrease in molecular weight of LDPE. the evaluation of the length of the lamellae for NR/LDPE composites is shown in Table 7.

Moreover, the table shows that with an increase in the rubber content, the length of the PE lamellae increases. For the LDPE sample, there was an increase in the lamella length by 8%, whereas for the LDPE/NR samples, a decrease in the chain length was seen. This is an indirect sign of a decrease in the molecular weight of LDPE. So with 10% rubber, the length of the lamellae is reduced by 6%, and with 50% NR, by 32%.

## 4. Discussion

Natural rubber has a significant effect on the structure and properties of LDPE, primarily fulfilling the main task—providing biodegradation of compositions based on a large-tonnage polymer when composted into the soil.

Structure formation in mixtures of thermodynamically incompatible polymers, such as polycrystalline polyethylene and amorphous rubber, occurs at the micro- and macrolevels [65]. At the macro level, depending on the ratio of the initial components, viscosity, molecular weight distribution and mixing technology, a coarse or highly dispersed system or an interpenetrating structure of a polymer mixture is formed. As a rule, with a small content of one of the components (no more than 10–25 vol.%), a dispersed structure of the mixture is formed. With increasing concentration, an interpenetrating structure is observed and its concentration region (phase reversal region) is determined by thermodynamics and mixing kinetics, as well as the ratio of the viscosity of the components, which we observed and which was confirmed by microscopy of LDPE/NR samples.

Differential scanning calorimetry have been used to analyze the influence of blend constituents and processing conditions on the compatibility, crystallization kinetics and the final crystalline content. A significant effect of NR on the process of LDPE crystallization was found. The melting temperature values remained almost constant, which could be explained by the similar size of LDPE crystallites, which melt at the same temperature. However, the enthalpy of melting and crystallinity of LDPE decreased noticeably with an increase in the NR content. Most likely, this is due to the violation of the flexibility of PE polymer molecular chains, which will lead to a decrease in the degree of crystallinity [66]. It was shown in [67,68] that the formation of miscible rubber compounds slows down the rate of crystallization when one of the components crystallizes. The absence of a change in the melting temperature also indicates that dispersion interactions between LDPE/NR are not dependent on the amount of NR [69]. Thus, in LDPE/NR composites, mutual diffusion between molecular chains is observed with the formation of interfacial regions [70]; however, this diffusion is very small, which leads to a decrease in the physical and mechanical properties of the material. The smallest diffusion is observed for the LDPE/NR 90/10 mixture, where the rubber was found in the form of large conglomerates, and the greatest diffusion for the 70/30 and 50/50 mixtures.

It should be noted that in reality the properties of elastomer blends rarely follow a linear or predictable correlation with the individual components [71]. It is likely that the mixing of amorphous and crystalline polymer causes a decrease in the mechanical properties of the entire system [69]. The tensile strength of the composite is reduced by more than two times with the introduction of natural rubber, changing linearly. However, the elongation of composites varies non-linearly, having a minimum value—an 80% lower decrease than that of polyethylene with a minimum rubber concentration of 10% wt., due to the clear phase separation boundary of the two polymers (Figure 3b), which leads to a break of the integrity of the system with less loads. As the polymer phases are homogenized (Figure 3c,d), the elongation of the material increases by 25%, which is consistent with the assumption of a negative contribution of the polymer phase interface to mechanical properties. Nevertheless, the mechanical properties of the obtained composites are suitable for use in various fields.

A significant increase in the contact wetting angle by more than 35% suggests the contribution of hydrophilic rubber to the properties of composites based on hydrophobic polyethylene. Therefore, the composites are highly hydrophilic, which allows micromycetes of the soil microbiota to gain a foothold on the surface of the films, initiating biodegradation. Probably, the nature and degree of homogeneity of the distribution of natural rubber in the LDPE matrix determines to a large extent significant changes in the hydrophobicity of the composite surface.

All this makes NR/LDPE composites suitable for applications in agriculture, packaging and other areas as an eco-friendly alternative to existing films and covering materials.

## 5. Conclusions

At present, polymeric materials are used in almost all areas of science and technology, in industry, agriculture, construction, etc. The huge scale of industrial production and a wide range of applications of polyolefins, including polyethylene, determine the importance of developing new materials based on them. The main and long-term direction in the development of new polymeric materials is to combine polymers with various substances and with each other in order to obtain materials with new required properties. However, mixing two materials does not simply add up their properties. The properties of multicomponent materials, as a rule, are very difficult to predict based on the composition and conditions of their preparation. If earlier the main advantage of synthetic polymeric materials was their durability, then over time it became clear that in the future such materials pose a serious threat to the environment. The main field of application of polyethylene is the manufacture of products with a short service life—packaging materials and agricultural products that turn into municipal solid waste before they lose their consumer properties and are characterized by high resistance to environmental influences, creating a serious environmental problem. One of the ways to solve the problem is the creation of polymeric materials, the utilization of which is possible under the influence of the microbiota of the environment. For this purpose, this article discusses compositions with the use of natural rubber as additives to synthetic polymers, which is a nutrient medium for microorganisms.

The effect of the additive of natural origin, natural rubber, on the structure and properties of synthetic polyolefin LDPE was shown in the work. LDPE and NR represent a thermodynamic complex system of two polymers: crystalline and amorphous, which significantly affects the properties and formation of the supramolecular structure of composites. It was found that natural rubber does not significantly reduce the physical and mechanical characteristics of composites; however, it allows biodegradable film materials to be obtained. Biodegradation is achieved by increasing the hydrophilicity of the material, which allows micromycetam to effectively populate the surface, initiating the process of biodegradation during composting into the soil. NR is also a substrate for more active development of microbiota on the surface of the composite. In the work, it was confirmed that biodegradation occurs not only in the NR phase, but also in the LDPE phase, due to a decrease in the length of the LDPE lamellae. This aspect of the work will be verified by the gel permeation chromatography method to assess the actual change in the molecular weight of the samples. Film materials occupy the first place among polymeric products for agricultural purposes. Significant interest in the use of mulching films for the purpose of killing weeds, retaining moisture, fertilizer, providing a better microenvironment for plants and protection from adverse climatic conditions has led to a rapid growth in the agricultural plastic film market and, as a result, the problem of processing tons of accumulated agricultural plastic waste. Today, this work opens up prospects for the creation of biodegradable film materials for agriculture, packaging and other areas of light industry.

## Figures and Tables

**Table 1:** Compositions of the LDPE/NR blends.

Sample	LDPE wt. %	NR wt. %
LDPE 100	100	0
LDPE/NR 90/10	90	10
LDPE/NR 70/30	70	30
LDPE/NR 50/50	50	50

**Table 2:** The size of NR conglomerates in the LDPE matrix depends on NR.

Sample	Average Value, Microns
LDLDPE 100	0
LDLDPE/NR 90/10	34.9
LDLDPE/NR 70/30	10.2
LDLDPE/NR 50/50	0.6

**Table 3:** Average values of the characteristics of the non-woven materials based on LDPE with different content of NR.

Sample	Tensile Strength (±0.5 MPa)	Relative Extension, (±10%)
LDPE 100	15.1	610
LDPE/NR 90/10	6.3	120
LDPE/NR 70/30	6.4	330
LDPE/NR 50/50	3.9	330

**Table 4:** The value of the contact angle depending on the content of NR.

Sample	Contact Angle, Deg. (±1 Deg.)
LDPE 100	79
LDPE/NR 90/10	72
LDPE/NR 70/30	65
LDPE/NR 50/50	50

**Table 5:** Differential scanning calorimetry of the initial samples.

Samples	*T*_m_, °C	Δ*H*, J/g	*χ*, %	*T*_m_, °C	Δ*H*, J/g	*χ*, %	*T*_m_, °C	Δ*H*, J/g	*T*_m_, °C	Δ*H*, J/g
	First Heating			Second Heating			First Cooling		Second Cooling	
PE 100	105.7	65.2	22.2	105.0	57.0	19.5	90.7	93.2	90.8	83.2
LDPE/NR 90/10	104.1	60.4	20.6	105.0	53.4	18.2	91.2	76.2	91.1	76.7
LDPE/NR 70/30	105.1	47.1	16.1	104.7	42.7	14.6	91.4	45.6	91.3	46.4
LDPE/NR 50/50	105.5	37.9	12.9	104.2	24.6	16.4	91.2	32.3	91.2	33.7

**Table 6:** Differential scanning calorimetry of the samples after exposure in the soil.

Samples after Exposure in the Soil	*T*_m_, °C	Δ*H*, J/g	*χ*, %	*T*_m_, °C	Δ*H*, J/g	*χ*, %	*T*_m_, °C	Δ*H*, J/g	*T*_m_, °C	Δ*H*, J/g
	First Heating			Second Heating			First Cooling		Second Cooling	
LDPE 100	107.3	74.59	25.46	104.8	60.41	20.62	91	80.79	90.8	89.05
LDPE/NR 90/10	105.1	66.87	22.82	105	53.79	18.36	90.7	75.36	91.1	76.06
LDPE/NR 70/30	103.7	56.66	19.34	104.7	49.9	17.03	91.3	62.02	91.3	57.31
LDPE/NR 50/50	105.4	55.52	18.95	104.2	48.17	16.43	91.5	61.35	91.4	57.2

**Table 7:** Changing the length of the lamellae after 24 months of exposure in solid.

Sample	Initial	After Exposure in the Soil
LDPE 100	0.0071	0.0077
LDPE/NR 90/10	0.0078	0.0073
LDPE/NR 70/30	0.0104	0.0081
LDPE/NR 50/50	0.0131	0.0089

## Data Availability

The data presented in this study are available on request from the corresponding author.
